# Quantifying precision and accuracy of measurements of dissolved inorganic carbon stable isotopic composition using continuous-flow isotope-ratio mass spectrometry

**DOI:** 10.1002/rcm.6873

**Published:** 2014-04-01

**Authors:** Susan Waldron, E Marian Scott, Leena E Vihermaa, Jason Newton

**Affiliations:** 1 School of Geographical and Earth Sciences, University of GlasgowGlasgow, G12 8QQ, UK; 2 School of Mathematics and Statistics, University of GlasgowGlasgow, G12 8QQ, UK; 3 Scottish Universities Environmental Research Centre (SUERC)East Kilbride, G75 0QF, UK

## Abstract

**RATIONALE:**

We describe an analytical procedure that allows sample collection and measurement of carbon isotopic composition (δ^13^C_V-PDB_ value) and dissolved inorganic carbon concentration, [DIC], in aqueous samples without further manipulation post field collection. By comparing outputs from two different mass spectrometers, we quantify with the statistical rigour uncertainty associated with the estimation of an unknown measurement. This is rarely undertaken, but it is needed to understand the significance of field data and to interpret quality assurance exercises.

**METHODS:**

Immediate acidification of field samples during collection in evacuated, pre-acidified vials removed the need for toxic chemicals to inhibit continued bacterial activity that might compromise isotopic and concentration measurements. Aqueous standards mimicked the sample matrix and avoided headspace fractionation corrections. Samples were analysed using continuous-flow isotope-ratio mass spectrometry, but for low DIC concentration the mass spectrometer response could be non-linear. This had to be corrected for.

**RESULTS:**

Mass spectrometer non-linearity exists. Rather than estimating precision as the repeat analysis of an internal standard, we have adopted inverse linear calibrations to quantify the precision and 95% confidence intervals (CI) of the δ^13^C_DIC_ values. The response for [DIC] estimation was always linear. For 0.05–0.5 mM DIC internal standards, however, changes in mass spectrometer linearity resulted in estimations of the precision in the δ^13^C_VPDB_ value of an unknown ranging from ± 0.44‰ to ± 1.33‰ (mean values) and a mean 95% CI half-width of ±1.1–3.1‰.

**CONCLUSIONS:**

Mass spectrometer non-linearity should be considered in estimating uncertainty in measurement. Similarly, statistically robust estimates of precision and accuracy should also be adopted. Such estimations do not inhibit research advances: our consideration of small-scale spatial variability at two points on a small order river system demonstrates field data ranges larger than the precision and uncertainties. However, without such statistical quantification, exercises such as inter-lab calibrations are less meaningful.

The carbon isotopic composition (δ^13^C value) of dissolved inorganic carbon (DIC), the δ^13^C_DIC_ value, has long been of interest to limnologists and oceanographers due to the insight that such measurements can provide on the source of inorganic carbon or processes that can affect the DIC pool. For example, the study of the changes in δ^13^C_DIC_ values in Loch Ness reveals the importance of heterotrophic utilisation of the dissolved organic carbon pool to the lake carbon cycle^1^; the growing use of stable isotope analysis in food web studies necessitates better understanding of compositional variation in baseline algal resources, thus the δ^13^C_DIC_ value has been measured to assess its significance in controlling composition.^2^ In addition, increasing focus on the carbon cycle and perturbations to this cycle enhance the significance of direct measurements of the DIC content and the δ^13^C_DIC_ values of water bodies. For example, direct measurement of the DIC concentration in lakes and river systems, accompanied by other parameters controlling solubility (e.g.,^3^), allows an assessment of whether these systems are ‘super-saturated’ with respect to the atmospheric equilibrium concentration and thus predominantly a source of CO_2_ to the atmosphere (e.g.,^4^); recently, the use of the δ^13^C_DIC_ value as a process fingerprint has been developed to estimate how much CO_2_ has been degassed from fluvial systems.^5^

Akin to many other isotope techniques, the manner in which we analyse water samples for their δ^13^C_DIC_ values has evolved with technological advances. The advent of high-frequency on-site measurements (e.g.,^6^,^7^) reveals detail through direct measurement, previously not possible due to the high sample volume. However, there will still be the need for manual sampling, e.g., expedition fieldwork in remote regions (e.g.,^8^), where transportation of equipment is not possible and sustained power may be a problem. Traditionally, measurement of δ^13^C_DIC_ values has required the collection of large sample volumes (to produce sufficient CO_2_ by use of vacuum line purge and trap followed by dual inlet mass spectrometry (e.g.,^9^,^10^)) and the use of toxic chemicals to stop microbial activity compromising sample characterisation (e.g.,^11^,^12^). Collection, storage and processing of samples for hydroecological studies may present logistical challenges that can preclude the widespread application of DIC characterisation. For example, the recovery of many large volume samples from remote areas is expensive, and challenging field conditions can reduce manual dexterity such that adding a toxic microbial inhibitor is difficult and this process is off-putting. Further, sample integrity could be compromised later in the laboratory during the transfer of the sample from the field collection bottle to the analytical vessel by gas loss (if the sample was over-saturated with respect to atmospheric conditions) or atmospheric contamination (if the sample was under-saturated). Precipitation of DIC as carbonate and subsequent analyses of this precipitate may have overcome some of these logistical problems (a review of this approach can be found in Atekwana and Krishnamurthy^13^), but the volume of water required for the reaction can still be large and further sample manipulation through filtering, drying and acid-evolution of CO_2_ is required. Continuous-flow isotope-ratio mass spectrometry (CF-IRMS) simplifies this approach and reduces analysis time. Advances made through the measurement of δ^13^C_DIC_ values by CF-IRMS (summarised in Table 1) include reducing the required sample volume and faster sample throughput (not given in Table 1 as of a similar order of magnitude, *ca* 10 min per sample by all authors).

**Table 1:** Summary of recently published approaches to measurement of δ^13^C_DIC_ values using continuous-flow isotope-ratio mass spectrometry. The ISO-CADICA system^6^ has been included here for completeness, although linearity effects are documented

Ref.	Concentration range	Sample volume	Calibration standard matrix	Internal standard precision
10	100–500 ppm CO_2_ (∼8–42 mM)	0.05–1 mL (concentration dependent)	Comparison with reference gas of known composition	± 0.5‰
11	∼2.5 mM	0.5 mL	Reference gas calibrated independently and internal DIC standard run	≤0.15‰
15	1–25 mM	1–5mL (2 ×12 mL field sample)	Same as samples	≤ 0.1 ‰
16	0.1–2.2 mM	5.9–100 mL	Solid or aqueous depending on the lab	0.1–0.5‰
21	0.6–5 mmol	0.9 mL (100 mL field sample)	Comparison to reference gas of known composition	≤ 0.2 ‰
27	3–60 ppm C (∼0.25–5 mM)	1–25 mL (concentration dependent)	Used contemporaneously run DOC standards previously calibrated by EA-IRMS	≤0.2‰
29	2–20 mmol	0.5–2 mL (50 mL field sample)	Routine running calibrated from carbonate standards	0.18‰ SD (n = 50)
30	0.4–8.1 mM	0.1–1.5 mL (concentration dependent)	Routine running calibrated from calcite standards	0.1‰
31	0.5–2.25 mM	300 μL min^–1^ (mobile phase) and 50 μL min^–1^ (acid)	Comparison with a pre-calibrated internal standard of a similar matrix and a reference gas	0.06‰
32	2–15 mmolal ∼2–15 mM	0.2 mL	Sodium bicarbonate or sodium carbonate standard solutions gravimetrically prepared	±0.1‰
6	0.1–2.8 mM	14.5 mL	Comparison of raw cavity ring-down spectrometry response with headspace analysis	>0.3 mM: ±0.1‰; <0.2 mM; <±0.3‰ Linearity caused 0.5‰ change

However, the DIC concentration for test standards in most of the studies documented in Table 1 is generally higher than that of many freshwaters, particularly those of low nutrient, good ecological status (e.g.,^14^). For samples of these low concentrations, mass spectrometer non-linearity presents an additional analytical challenge. Such non-linearity has been noted by other authors of DIC methodological studies (e.g.,^11^,^15^),but is rarely discussed in depth as often the analyte concentration has usually been sufficiently large that the machine response has been linear. Here, using a field-to-laboratory approach for collection and measurement of DIC and δ^13^C_DIC_ values, we consider mass spectrometer linearity and estimate the precision and accuracy of an unknown measurement. They are quantified by calculating the standard deviation on the measurement on an unknown, s_0_ (precision), and the 95% confidence intervals, CI (accuracy). This approach is quite different from the normally reported measurement of precision as being the repeat measurement of an internal standard or field matrix sample (e.g.,^16^), but it is critical that the community considers it.

## EXPERIMENTAL

### Pre-sampling preparation

All sample analyses were carried out in 12 mL screw-capped glass vials (Exetainer®, Labco, Lampeter, UK) hereafter referred to as exetainers. All vials were acid-washed prior to use (24 h in 5 M HNO_3_), rinsed with distilled water and oven-dried at 60°C. A volume of 150 μL of 103% phosphoric acid was pipetted into each exetainer, the cap replaced and the acid-filled exetainer evacuated for 1 h by being attached to a vacuum line specially adapted with syringe needle fittings to pierce the septum. Although the same acid-washed exetainers were used repeatedly, a new septum was used for each new use.

### Sample collection

Field samples were collected from the Glen Dye catchment, a headwater subcatchment of the River Dee in Aberdeenshire, NE Scotland. Samples were collected at two different catchment scales: from Brocky Burn, a second order stream draining 1.3 km^2^, and from the Water of Dye (at Bogendreip Bridge), a third order stream draining 90 km^2^. Brocky Burn drains a relatively pristine peatland, with few trees in the riparian zone, while there is a modest riparian zone, with birch (*Betula* spp.) and Scots pine (*Pinus sylvestris*) present, and marginal pastoral land and some forest plantation in the catchment at Bogendreip. A fuller description of the field site can be found in Soulsby *et al*.^17^ The δ^13^C_DIC_ values and the [DIC] were hydrologically sensitive, ranging from –22.0 to –4.9‰ and 0.012 to 0.468 mM C under different flow conditions.^14^

Samples were collected as follows. At each sampling point, the river was sub-sampled using a 10 L plastic bucket, which was rinsed three times with river water prior to collecting the aliquot for DIC analysis. A 10 mL plastic syringe and disposable needle were rinsed three times in the flowing river water, and then usually filled to 10 mL underwater in the plastic bucket. Filling underwater was repeated if bubbles were evident in the syringe barrel. While maintaining the syringe underwater within the beaker, the sample was introduced into the exetainer by piercing the septum. Due to pre-evacuation of the exetainer, in under 1 min, the syringe barrel was sucked in as the sample was transferred into the vial. Suction of the barrel was used as a quality control measure to indicate that the exetainer had retained vacuum and to instil confidence that the sample would be subject to minimal contamination from atmospheric CO_2_. It was unusual for the vacuum to fail in a given exetainer, and samples where the syringe barrel was not drawn fully were rejected (this averaged fewer than 1 per 45 samples). Although sample filtration is used in some DIC analytical approaches, we do not recommend this for field samples where the pH is less than 7.5, as a proportion of the inorganic C pool will be present as free CO_2_ and this could degas during filtration and so compromise the δ^13^C_DIC_ values and the [DIC].

Although the sample will degas when injected into the evacuated exetainer, the 10 mL filled exetainer may not be at atmospheric pressure. In this condition, air can be incorporated during removal of the syringe (a ‘sucking’ sound may be heard, and reproducibility between replicate measurements of the δ^13^C_DIC_ value and the [DIC] may be poor). Thus, in addition to filling the exetainer under water to avoid air ingression, we advocate removal of the syringe underwater, done holding the syringe where the needle joins the syringe barrel (to avoid separation from the barrel and further sample entering the exetainer), and then swiftly and smoothly withdrawing it from the septum. Removal of the syringe may allow sample ingression, which can result in poorer precision in measurement of the [DIC] (as the calibration is geared towards a constant headspace volume). However, if the water sampled is isotopically homogeneous, sample ingression should not lead to inaccuracies in the measurement of δ^13^C_DIC_ values, which was our primary aim in developing this technique.

After the 10 mL field sample had been introduced, the exetainer was hand-shaken to thoroughly mix the sample and the acid. The phosphoric acid reduces the pH and in doing so the DIC pool is converted into free CO_2(aq)_ and the resultant low sample pH, typically 2, inhibits microbial activity. The sample is stored upside-down with the liquid in contact with the septum, thus minimising headspace CO_2_ ingression or egression, and transported in this manner to the laboratory to await analysis. Triplicate samples were collected in the field, with two used for primary analyses and a third retained for subsequent analyses if there was poor agreement between the paired samples. A visual demonstration of this sample collection approach is available.^18^

### Sample analyses

Paired field samples considered here were generally analysed within 5 days of return to the laboratory (2 days of this were required to prepare standards for calibration of field samples). Measurement of δ^13^C_DIC_ values and the [DIC] were executed at SUERC using two different automated continuous-flow isotope-ratio mass spectrometers: (i) a Fisher Scientific Delta V Plus interfaced with a Gas Bench II sample preparation and introduction system (both from Thermo Fisher, Bremen, Germany), hereafter referred to as the Delta V-GBII system, and (ii) a VG Optima interfaced with an Analytical Precision (AP) gas preparation bench (neither supplier now exists).

**Figure 1:**
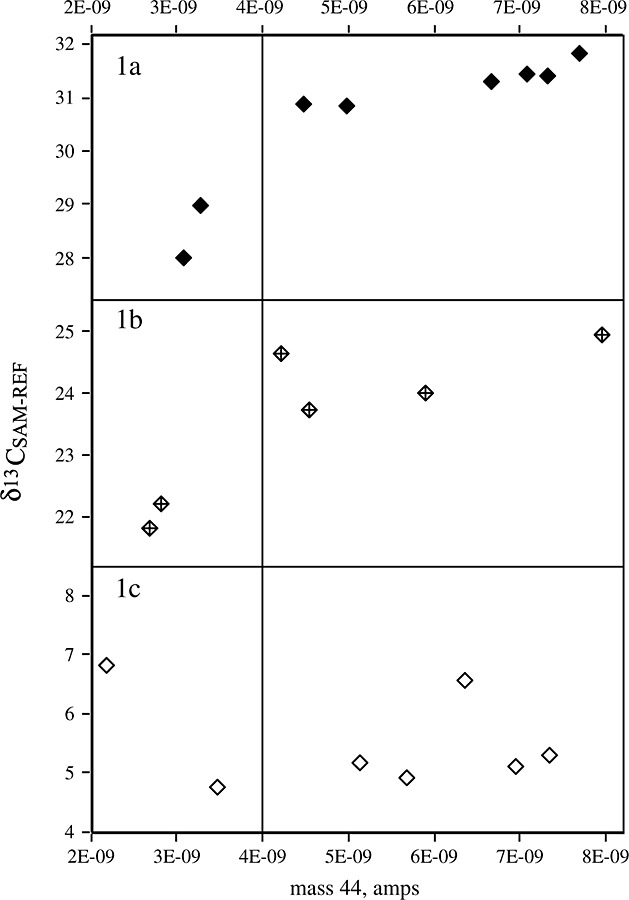
δ^13^C_SAM-REF_ values as a function of mass 44 intensity (amps), showing change in δ^13^C_SAM-REF_ with decreasing intensity. This data is from the Optima run named DIC 13 (Table 2).

The Delta V-GBII system was designed to be used in continuous-flow mode. The sampling and mass spectrometric analysis of the headspace gas are similar to procedures documented elsewhere (e.g., Fig. 1 in Yang and Jiang^19^) and so are not discussed further here. The analysis time is approximately 15 min. However, the Optima, purchased in approx.. 1991, had not been purchased for use in CF mode; thus, we manually introduced a glass capillary from the AP gas preparation interface to the source using an open split, with a ratio of ∼30:1 loss/intake to the mass spectrometer. This AP interface pre-dates, but functions analogously to the Gas Bench. We also modified the Optima software (version 1.55) to pressurise the exetainer headspace for 1 min. In addition, as we were working with low-concentration samples, the sample loop used in the AP gas preparation interface was seven times the normal size (700 μL) and to minimise mass spectrometer linearity effects, the reference gas pressure was reduced to a mass 44 intensity of ∼2.5e-9 amps. This approximated the beam intensity produced from a 0.25 mM sample (mid-range for our field samples) in 12 mL exetainers with 2 mL of headspace. One sample could be analysed in approximately 6 min.

**Table 2:** The precision on unknown, s_0_, for each calibration for δ^13^C_DIC_ values considered to be isotopically linear and non-linear, and for [DIC]. Data do not exist for each calibration as for some runs neither non-linear δ^13^C_DIC_ values or [DIC] needed to be calculated. The R^2^ values for all δ^13^C_DIC_ and [DIC] calibrations ranged from 98.6 to 99.9 and from 90.8 to 96.3, respectively. All calibrations were highly significant (*p*-values <<0.001) except for the non-linear DIC11 calibration, which was still significant (*p*-value = 0.017) but with a larger *p*-value as this contained fewer data points

Run	Linear δ^13^C_DIC_ s_0_			Non-linear δ^13^C_DIC_ s_0_			[DIC] s_0_		
Optima	mean	min	max	mean	min	max	mean	min	max
Dic5	0.37	0.36	0.39	0.61	0.58	0.66	0.0299	0.0284	0.0339
Dic6	1.11	1.08	1.18	0.86	0.82	0.97			
Dic6	0.42	0.41	0.47						
Dic8	0.32	0.31	0.33				0.0295	0.0276	0.0334
Dic9	0.64	0.63	0.67				0.0306	0.0280	0.0364
Dic10	0.37	0.36	0.40				0.0416	0.0400	0.0458
Dic11	0.27	0.26	0.29	0.74	0.68	0.86	0.0252	0.0240	0.0282
Dic12	0.62	0.60	0.66				0.0228	0.0215	0.0261
Dic13	0.56	0.55	0.59	1.15	1.10	1.27	0.0278	0.0262	0.0319
Dic14	0.56	0.54	0.61				0.0191	0.0179	0.0223
Dic15	0.69	0.68	0.71				0.0226	0.0211	0.0267
Dic16	0.58	0.58	0.60	1.60	1.57	1.66	0.0182	0.0176	0.0197
mean	0.54	0.53	0.58	0.99	0.95	1.08	0.027	0.025	0.030
median	0.56	0.54	0.60	0.86	0.82	0.97	0.0265	0.030	0.030
SD	0.23	0.22	0.24	0.39	0.40	0.39	0.007	0.007	0.008

For calibration, it is generally considered more rigorous to use standards as similar in composition to the analyte, and is particularly appropriate in analytical approaches where isotopic fractionation occurs – there is a liquid-headspace fractionation associated with DIC analysis.^15^ Thus, to avoid error in correcting for liquid-headspace fractionation,^16^ and as we were concerned that values of δ^13^C are influenced by mass spectrometer non-linearity, we prepared aqueous DIC standards of different concentrations for calibration through linear regression. The range in concentration also allowed us to calibrate for [DIC]. We used three isotopically distinct sources of inorganic carbon (one NaHCO_3_ and two CaCO_3_, sourced in-house using Fisher Scientific and BDH Chemicals). We defined their δ^13^C values by a modification of the phosphoric acid method,^20^ namely temperature-controlled reaction with excess H_3_PO_4_ in a helium-flushed vial, followed by gas separation and measurement of δ^13^C_V-PDB_ values using an AP2003 mass spectrometer. The standard deviation of repeated measurement of the δ^13^C values of these internal standards is better than ±0.1‰. Importantly, for statistical purposes, the range in the measured δ^13^C_VPDB_ values of the standards was greater than the field range for which this technique was developed, ∼ –5‰ to –22‰.^14^

As we were using internal inorganic carbon standards to calibrate an unknown sample, the δ^13^C value of the CO_2_ reference gas (which came from a cylinder) was relatively unimportant, rather only constancy in reference gas composition during the analytical process was required. Constancy of reference gas composition was assured by measuring a fourth internal inorganic carbon standard (a Ca/Na-CO_3_ mixture, manufactured by bubbling CO_2_ through a NaOH solution, hereafter known as the ‘drift internal standard’) after every 10^th^ sample.

The [DIC] of the field samples was unknown; thus, in the initial periods of the field campaign for which this technique was developed, additional sacrificial samples were collected to assess the concentration range requiring to be calibrated. For this field site, the [DIC] ranged from 0.05 to 0.5 mM,^14^ typical of field areas poor in carbonates. To avoid compromising the internal aqueous standard composition (by loss or gain of CO_2_) we did not make up one large batch of each standard and sub-aliquot this batch, but rather created each standard individually. There are elegant descriptions of semi-automated approaches to aqueous standard preparation (e.g.,^15^,^21^) but demand on similar pre-sample preparatory interfaces is such that to accommodate swift turnaround after field collection, we manually prepared the calibration standards.

For concentrations of 0.05–0.5 mM in a 10 mL sample, aliquots of inorganic carbon less than 0.5 mg were weighed to create each standard using a MX5 microbalance (Mettler Toledo, Greifensee, Switzerland), precise and accurate to 0.001 mg. The range of the balance precluded weighing directly into the heavier exetainer; rather, the aliquots were weighed into small acid-washed glass buckets, the contents tipped into the exetainer, and the bucket reweighed to calculate the mass transferred. As with the preparation for field sampling, post standard addition, the exetainers were evacuated for 1 h, after which 10 mL of boiling water, acidified to pH 1 by the addition of degassed H_3_PO_4_ (approximately 10% by volume), was introduced using a disposable syringe. Boiling water, used to ensure minimal blank and to enhance dissolution of the CaCO_3_ standards, negated the possibility of filling the samples under water. The aqueous internal standards were then mixed for 10 s by hand-shaking. The field samples were turned upright and similarly mixed for 10 s. The standard-sample ‘run’ was arranged in analytical sequence: two sacrificial conditioner samples to ensure that the system was functioning properly, then internal aqueous calibration standards and finally samples randomly ordered, with the drift internal standard every 10 samples. The analysis-ready sample sequence was left to equilibrate for 24 h in the temperature-controlled room, a period identified^11^,^15^,^21^ to be sufficient to allow headspace-liquid equilibration to occur. Blanks were prepared using the above protocol, but without the addition of an internal standard. The standard preparation procedure has also been recorded for visual reference.^18^

### Treatment of data and statistical analyses

Statistical analyses was carried out using Minitab 15 Statistical Software (2007) (Minitab Inc., State College, PA, USA) using a macro, V15. Our approach to generating the precision of a measured unknown is as follows:

For each analytical run, the value of δ^13^C relative to the reference gas, δ^13^C_SAM-REF_, for each internal aqueous calibrant standard was plotted against the area of mass 44 (Delta V- GBII) or the mass 44 intensity (the Optima). This was to visualise the linearity of the relationship, considered ‘mandatory’ in assessing data responses.^22^ From this assessment, it was apparent that generally with decreasing beam intensity the δ^13^C_SAM-REF_ value changed. An example typical of this response for the Optima is shown in Fig. 1. However, the change in composition was not consistent in size or direction with the three different standards – both increases and decreases in ^13^C-content occurred. Such inconsistency in direction and magnitude has been noted by others (e.g.,^15^). Thus, pooling standards of different sizes across the concentration range without having first established if there is a non-linearity effect is an incorrect approach, although likely to give rise to a linear response if the range is sufficiently large.

Our experience with observing non-linearity on multiple runs suggested that the trend was towards a common value (termed here the process blank to encompass mass spectrometer and standard formation contributions). It is possible to mathematically correct for such a process blank if the blank composition can be defined. The blank δ^13^C value and size can be estimated from plotting the δ^13^C_SAM-REF_ value as a function of inverse mass 44 intensity with the blank composition determined from where the regressions for each internal standard intercept. However, we did not find that all the standard regressions intercepted, indicating that changes in the δ^13^C_SAM-REF_ value resulted from factors more dominant than a process blank contribution. Thus, to correct an unknown for a poorly constrained blank would introduce error and create greater inaccuracy than correcting for non-linearity in δ^13^C measurement through use of multiple calibration lines, which incorporate the influence of a process blank.

For all analytical runs, from x-y plots of δ values as a function of sample size (area 44; mass 44 intensity), data ranges were identified where the mass spectrometer response was considered linear, or non-linear, termed ‘mass-spec linear calibration’ and ‘mass-spec non-linear calibration’, respectively. The cut-off between these responses was chosen to be closer to the linear than the non-linear response, thus ensuring larger confidence intervals were attached to unknown responses. This approach is discussed further later.

**Figure 2:**
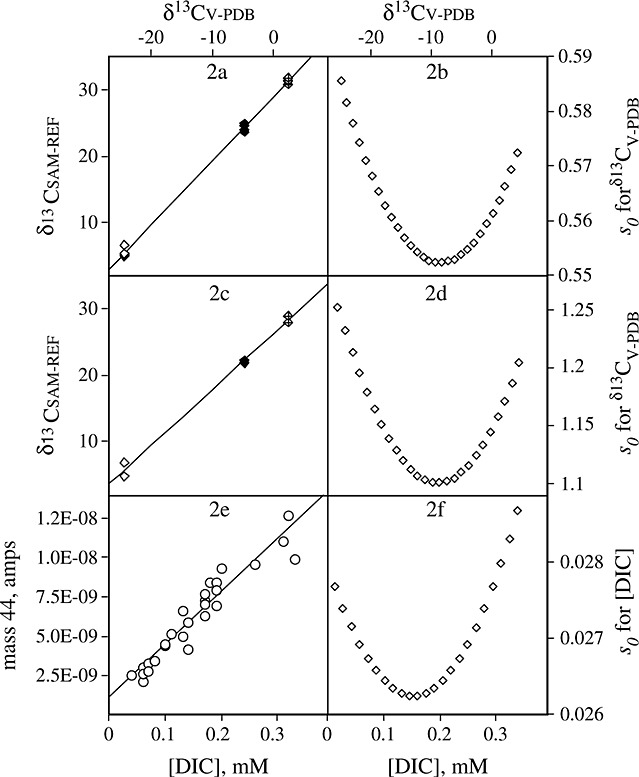
(a, c and e) Data used to calibrate for linear and non-linear δ^13^C_DIC_ values and [DIC], respectively. All data are from the same analytical run, DIC13 (Table 2), chosen as it approached mean responses for each variable given in Table 2. (b, d and f) Depiction of s_0_ corresponding to the adjacent linear regression.

Ordinary least-squares linear regressions were carried out for all calibrations, regressing the δ^13^C_SAM-REF_ value as the response variable y upon the δ^13^C_VPDB_ value (e.g., Figs. 2(a) and 2(c) which uses data from calibration DIC 13 on the Optima). Inverse regression (or calibration)^23^ was then carried out to provide estimates and uncertainties of the unknown samples using a Minitab *Calibration* or *Inverse Regression* macro.^24^ Linearity effects in concentration were not prevalent with either mass spectrometer, and thus the calculated [DIC] for all calibration standards (calculated from the recorded weight used to form each standard) was plotted against mass 44 (Fig. 2(e)). The linear regressions were unweighted^25^ as the errors on the y and x variables are considered to be of a similar magnitude. Using this approach we could check for linearity^25^ through examination of the residuals from the regression line, calculate the standard deviation^26^ (s_0_) of δ^13^C_DIC_/[DIC] for an unknown field sample, and calculate the 95% CI widths for an unknown value of δ^13^C_DIC_.

Unlike the samples, DIC standards were created in the exetainer and not filled underwater, and so air ingression is possible and the δ^13^C values and [DIC] may be compromised. In addition, errors in weight may affect the [DIC]. To assess whether the difference between replicate field samples is greater than the uncertainty on an unknown we compared s_0_ of an unknown (generated through calibration) with a measure of the variation in paired sample replicate composition. This is necessary to decide whether duplicate field sample analysis was necessary. The paired replicates were field samples collected over a 15-month field survey period^14^ spanning the range of concentrations and isotopic compositions of these calibrations, and for assessment over the full range, the δ^13^C_DIC_ values generated by linear and non-linear calibrations were pooled.

## RESULTS AND DISCUSSION

The high values of R^2^ (Table 2) suggest there is little deviation from linearity in the relationships between δ^13^C_SAM-REF_/area response and δ^13^C_DIC_/[DIC], respectively. In addition, examination of the residuals for each of the calibration lines generally showed a random pattern, and the residuals showed a normal distribution, representative of a strong linear relationship between the response variables, the δ^13^C_SAM-REF_ value and mass 44 area. Calibration by linear regression is thus an appropriate approach in this method to estimate the δ^13^C_DIC_ and [DIC] values of an unknown.

To quantify precision on an unknown, e.g., a field sample, we calculate s_0_, the standard deviation of the unknown. This requires reconstruction of the error on a given x value from the y value, which is itself is subject to uncertainty s_y_, the standard error of estimate for a given value in the calibration line.^22^ In brief, this assessment^23^ incorporates components additional to the uncertainty on the response y, such as the number of data points in the calibration and the fit of the line, and thus generally produces greater uncertainties on the precision of an unknown sample than s_y_ (but the latter is more commonly quoted). Figures 2(b), 2(d), and 2(f) show the corresponding s_0_ values for the calibrations shown in Figs. 2(a), 2(c), and 2(e). The curved shape indicates that the values of s_0_ are not equal for the data range, but typically increase with increasing distance from the centre of the data range as the unknown value becomes less well-constrained.

The standard deviation of the unknown, s_0_, ranges from 0.22 to 1.18‰, and from 0.58 to 3.22‰, for linear and non-linear δ^13^C_DIC_ calibrations, respectively (Tables 2 and 3), and from 0.0176 to 0.0458 mM for [DIC] calibrations (Table 2). The non-linear δ^13^C_DIC_ calibrations have larger s_0_ values than the linear δ^13^C_DIC_ calibrations (Table 2). This can occur for two reasons. First, a larger range in δ^13^C_SAM-REF_ values is likely with mass spectrometer non-linearity. Secondly, the calculation of s_0_ is influenced by the number of replicates of an unknown and so calibrations with fewer replicates, when all else is equal, will have a larger s_0_ value. For example, the non-linear calibrations for the Delta V-GBII contained fewer replicates of each standard than the non-linear calibrations for the Optima and so the s_0_ value is generally larger. For this reason, the non-linear Delta V-GBII calibrations are not included in the general discussion of the typical range of s_0_.

**Table 3:** The precision on unknown, s_0_, for δ^13^C_DIC_ calibrations using all data, and responses considered to be isotopically linear and non-linear (identified as >/< mM concentration, respectively, in the run name). The larger uncertainties associated with the Delta V-GBII than with the Optima largely arise as the calibrations in the non-linear range comprised fewer replicates of each standard and so the prediction intervals are wide and the corresponding values of s_0_ larger than would be expected if more replicates has been included. In addition, for these datasets the maximum 95% confidence interval (CI) widths for each calibration have been calculated. This is a measure of the accuracy of the measurement, representing the ± ‰ range of the true δ^13^C_DIC_ value that 95% of an unknown sample population will be within

Run name	δ^13^C_DIC_ s_0 max_	δ^13^C_DIC_ s_0 min_	δ^13^C_DIC_ s_0 max_	δ^13^C_DIC_ s_0 min_	δ^13^C_DIC_ s_0 max_	δ^13^C_DIC_ s_0 min_	±95% CI		
	All		Linear		Non-linear		All	Linear	Non-linear
DIC120116 >/< 0.30 mM	1.05	1.00	0.42	0.38	1.22	1.14	2.23	0.98	2.92
DIC120202 >/< 0.40 mM	0.71	0.68	0.22	0.20	0.70	0.65	1.51	0.54	1.60
DIC120120 >/< 0.40 mM	0.81	0.78	0.32	0.30	0.87	0.79	1.70	0.76	1.92
DIC120203 >/< 0.40 mM	0.86	0.82	0.46	0.42	0.81	0.75	1.81	1.05	1.85
DIC120112 >/< 0.40 mM	0.84	0.81	0.30	0.28	0.88	0.82	1.80	0.74	2.03
DIC110705 >/< 0.40 mM	2.63	2.55	0.96	0.83	3.22	3.12	5.61	2.57	7.15
DIC110707 >/< 0.28 mM	1.30	1.28	0.51	0.49	2.05	1.86	2.73	1.08	5.00
DIC110815 >/< 0.28 mM	0.90	0.85	0.34	0.29	0.94	0.86	1.95	0.91	2.18
mean	1.14	1.10	0.44	0.40	1.33	1.25	2.42	1.08	3.08
median	0.88	0.835	0.38	0.34	0.91	0.84	1.88	0.95	2.11
SD	0.63	0.62	0.23	0.20	0.87	0.85	1.34	0.63	1.97

Using the Delta V-GBII data as an example, we calculated the 95% CI applicable to an unknown sample and summarised their half-widths. The half-widths define the range either side of the calibration line within which 95% of the population is likely to lie and so can be considered an estimate of how accurately we can define an unknown composition. The calculated half-widths of the 95% CI range from ±0.54‰ (linear calibration) to ±7.15‰ (non-linear calibration), although the maximum median widths of the 95% CIs ranged from ±0.94 to ±2.11‰ (Table 3). The 95% CIs are wider on the non-linear datasets than the linear datasets due to the greater uncertainty associated with s_0_. In turn, inclusion of the non-linear data in the full dataset renders the 95% CI widths more comparable with the non-linear estimates.

**Figure 3:**
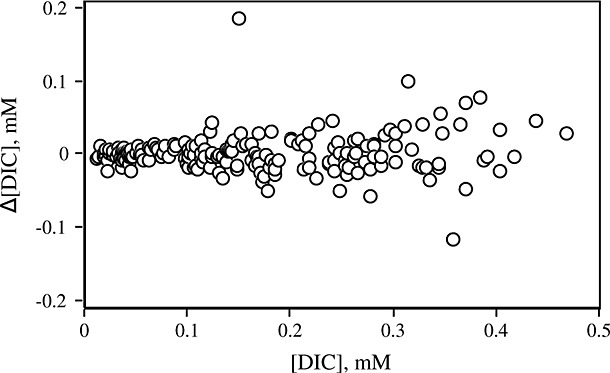
The [DIC] difference between paired unknowns as a function of [DIC] shows that increased pair-scatter is apparent with increasing [DIC].

From measurement of 199 paired duplicates (measured on the Optima) we calculate the mean difference ± standard deviation on that mean to be –0.01 ± 0.98‰ for δ^13^C_DIC_ values and 0.002 ± 0.026 mM for mM[DIC]. Consideration of the difference in values of δ^13^C_DIC_ or [DIC] between pairs over the ranges measured shows that increased pair-scatter is apparent as [DIC] increases (Fig. 3). However, such a systematic relationship is not observed with δ^13^C_DIC_, i.e., the same scatter in δ^13^C_DIC_ between pairs can be found over the range of field sample composition.

For lower concentration DIC sample characterisation, we chose linear regression to calibrate field sample δ^13^C_DIC_ values and [DIC], using internal standards of the same matrix as the unknown. Ultimately, the accuracy of measurement of our samples will depend on good calibration of the internal standards used to correct the δ^13^C values of the DIC standards, but as these were inherited from another laboratory, we do not investigate this aspect of accuracy. Rather we assume that the internal standard composition has been accurately measured and focus on discussion of the uncertainty of the precision in an unknown, calibrated using these standards.

### Why use linear regression?

For each required calibration there is a good linear relationship between predictor, δ^13^C_DIC_ value and response, the δ^13^C_SAM-REF_ value (Table 2 and comparison of residuals described earlier). This suggests that headspace equilibrium post-acidified sample collection is a valid method to estimate the δ^13^C_DIC_ value and [DIC]. Multiple standard calibration seems to be more prevalent in other approaches to measuring [DIC] by CF-IRMS, but only two other groups^6^,^15^ (Table 1) have utilised linear regression of aqueous DIC standards to correct values of δ^13^C_DIC_. St-Jean^27^ used linear regression (personal communication) but the system that he developed allows contemporaneous measurement of δ^13^C_DIC_ and δ^13^C_DOC_ values and thus he used DOC standards to avoid the problems of change in composition of DIC standards over time. Assayag *et al*.^15^ adopted linear regression to remove the need to correct for fractionation by headspace equilibration, a process prone to significant error when the headspace to liquid volume increases as the constants required to make this correction are not for waters of high ionic strength (typical of the field sample after treatment with acid to allow δ^13^C_DIC_ measurement by headspace CF-IRMS). We also adopted linear regression for the calibration of low-concentration samples, as the use of multiple standards spanning the isotopic composition of field samples goes some way towards correcting measurements of δ^13^C_SAM-REF_ values for differential responses of process linearity (compare Figs. 1(a) and 1(b) with 1(c)). After primary calibration, many laboratories (e.g.,^16^) only use a one-point calibration for data normalisation and this may not detect changes in either slope or intercept.

Linear regression also likely improves accuracy and precision. The use of multiple calibration standards corrects for inaccuracy resulting from inter-run changes in slope, or compression or extension of the calibration lines, that may not be noticed with only one internal check standard (e.g.,^16^). However, linear regression using multiple standards allows estimation of the standard deviation on the unknown, s_0_, and the half-width of these 95% CIs thus offers a truer representation of our ability to infer statistically significant differences between field samples or assess the limitations of a new technique. To date, studies investigating the utility of CF-IRMS for the measurement of δ^13^C_DIC_ values (Table 1, except^7^) have proposed that the standard deviation of the mean of repeat determinations of a known, or unknown, is representative of the ‘precision’ with which an unknown field sample can be measured, and the 95% CI widths are not documented. Whilst allowed by the community, this is an assumption and where data exists to more rigorously estimate the ‘precision’ and ‘accuracy’ of an unknown, or to generate that data, we suggest that a more rigorous approach to estimating precision and accuracy, such as that we outline here, should be taken.

### Comparison with other methodologies

All the published precision estimates for measurements of δ^13^C_DIC_ values by CF-IRMS, as ‘represented’ by standard deviations on the mean of a measured sample (Table 1), are less than s_0_, the standard deviation on an unknown sample composition (Table 2) and our estimate of precision. This is not surprising, since these two approaches to describing sample ‘precision’ are fundamentally different. The approach that we document estimates the precision in an unknown sample over the full calibration range, and is shaped by the errors in producing and measuring individual aliquots of calibration standards. Thus, this approach incorporates uncertainty from all possible effects associated with the methodology (except error in the assumption that the δ^13^C_VPDB_ value of the internal standards is correct) and is a more appropriate means to express how precise our measurements are. This estimate of ‘uncertainty’, s_0_, is not the same as an estimate of how well one sample can be measured repeatedly, i.e., the precision on a repeat measurement, possibly even generated by repeat analysis of the same internal standard, on the same analytical run. We present data from multiple runs showing inter-run differences. If we adopt a similar approach and estimate precision by calculation of the standard deviation of the mean of the internal control standard, our estimates of precision range from 0.16 to 0.41‰ (assessed over six different runs, n = 8–13 individual samples per run, [DIC] ∼0.15 to 0.25 mM), which is less than s_0_ (Table 2) and closer to the estimates of precision generated by others (Table 1) for samples of generally higher [DIC].

The 95% CI widths are larger than s_0_, with the smallest ranges observed where the Delta V-GBII response was linear and the largest ranges where the Delta V-GBII response was non-linear. For both s_0_ and the 95% CI widths, using all the data in the calibration gave rise to estimates more similar to the non-linear range. Thus, the greatest interpretative power arises from splitting the calibration to accommodate linearity. With the exception of DIC110705 and DIC110707 (Table 3), we can be more confident in the value of an unknown estimated from a linear calibration than that calculated from the larger dataset calibration. Conversely there is little reduction in confidence in a sample calculated from a non-linear range than from the whole dataset.

Given that our internal precision calculated as the standard deviation of the mean of the internal control is comparable with others (Table 1), it is reasonable to assume that if other labs quantified precision and accuracy as documented here, similar ranges in precision and accuracy would be calculated. This becomes important for quality control, for example in inter-laboratory calibration exercises where analysis of the causes of differences does not focus on normalisation procedures, but on sample processing, storage and matrix.^16^ To expand on this, if our lowest Delta V-GBII median 95% CI widths (±2.1) and s_0_ values (±0.4 ‰) represented accuracy and precision across the laboratories in the recent DIC inter-lab calibration,^16^ the interpretation that there were differences in the measurement of δ^13^C_DIC_ values of lake water and sea water between laboratories could not be made.

It is conceivable that the technique we have outlined might lead to larger estimates of s_0_ and 95 % CI than are observed in between-measurement variation: injection of liquid into the exetainer during standard preparation may allow gas ingression/egression and thus increase errors and generate greater s_0_ values than if standards were prepared in the same manner as field samples are collected, i.e. samples introduced below water. Duplicate data allow us to experimentally define the between-measurement variation, the principle being that all has remained the same, and that the duplicate is a repeat measurement (replicate) of all the processes which introduce variation.^22^ The closeness of the mean Δ^13^C_DIC_ and Δ[DIC] to zero for the 199 paired field samples indicated no bias in the collection and analysis of replicates, and from this we infer that where duplicate analysis is not possible, analysis of one sample may still provide representative data. However, the error on the measurement of offset between pairs is comparable with the unknown sample precision for both [DIC] (0.026 vs. mean s_0_ value of 0.027, Table 2) and δ^13^C value (0.98‰ for all data pooled vs. mean s_0_ values of 0.54‰ for linear and 0.99‰ for non-linear, Table 2). This suggests that error in internal standard production and analysis is comparable with error associated with natural standard collection and analysis, and/or that the field sample reservoir sub-sampled is not heterogeneous. The desire to collect field samples in an ‘analysis-ready’ manner precludes the use of automated volume control (manual injection in the field is used to fill the vial) and this intra-replicate volume difference may contribute to intra-replicate isotopic variation (discussed in Assayag *et al*.^15^).

To use this approach we needed to create a process sufficiently constrained for low-concentration DIC samples to be used successfully in field studies. Whilst our approach shows that at this concentration there can be mass spectrometric linearity effects, which when normalised reduce the precision with which we can measure an unknown δ^13^C_DIC_ value to between 0.5 and 3.2‰ depending on [DIC], this is still sufficiently small that field research is not undermined. For example, repeated sampling of the same three locations in the Glen Dye catchment shows that over a 24 h period, the δ^13^C_DIC_ values of small order rivers can show hydrologically induced changes of ∼18‰, exhibit diurnal variation of up to 8‰, or remain quite constant.^14^ Field data for two of these sampling points shows there to be variation in sample composition in a reach, either influenced by in-flow of an isotopically different source, or inferred to occur due to differences in the amount of photosynthetic activity (Table 4). For example, at Brocky, three of four sites in a 100 m reach show reasonable homogeneity in δ^13^C_DIC_ values, with the more ^13^C-depleted DIC sampled from the darkest point in the stream where respiration is probably more dominant and causes ^13^C-depletion of the DIC pool (e.g.,^28^). Similarly, at Bogendreip, over a 15 m reach, the δ^13^C_DIC_ value downstream of the field drain is lower than upstream of the field drain. We infer that this is due to the input of a ^13^C-depleted source, the field drain. In the field drain, light was excluded and thus respiration has generated ^13^C-depletion of the DIC pool. The concentration of these samples is sufficiently high that they would have been measured in the region where the mass spectrometer response is linear and the mean inverse 95% CI on values of δ^13^C_DIC_ approximates to 0.54‰. Most replicates show smaller paired offsets than this (Table 4), but the Δ[DIC] between replicates is comparable with s_0_ (Table 2).

**Table 4:** δ^13^C_DIC_ values and [DIC] of duplicate aliquots of a field sample collected at different nested catchment scales, Brocky, 1.3 km^2^, and Bogendreip, 90 km^2^, in the Glen Dye catchment, NE Scotland. The sites marked with an asterisk are part of a larger dataset^14^

Sample	Date/Time	δ^13^C_DIC_	δ^13^C_DIC_	Δ^13^C_DIC_	[DIC], mM	[DIC], mM	Δ[DIC], mM	Comments
Brocky 1	1/7/03 10.57	–7.3	–6.9	–0.4	0.132	0.123	0.009	At discharge gauge^*^
Brocky 2	1/7/03 11:00	–7.5	–7.1	–0.3	0.158	0.176	–0.017	10 m upstream from Brocky 1
Brocky 3	1/7/03 11:11	–7.3	–7.6	0.3	0.122	0.135	–0.012	At bifurcation in stream channel, ∼50 m upstream from Brocky 1
Brocky 4	1/7/03 11:20	–10.7	–10.8	0.1	0.141	0.154	–0.013	Sampled under west bank overhang of a 3 m diameter pool ∼100 m upstream from Brocky 1
Bogendreip 1	1/7/03 14:15	–5.2	–4.0	–1.3	0.186	0.181	0.006	3 m upstream of field drain
Bogendreip 2	1/7/03 14:10	–24.6	–25.1	0.4	0.405	0.383	0.021	At field drain outflow
Bogendreip 3	1/7/03 14:05	–6.4	–6.7	0.3	0.210	0.164	0.045	1 m downstream of field drain
Bogendreip 4	1/7/03 14:00	–6.5	–6.2	–0.4	0.187	0.182	0.005	5 m downstream of field drain^*^

This field study demonstrates that the natural variation in δ^13^C_DIC_ values is sufficiently large that the loss of precision that occurs due to mass spectrometer non-linearity when analysing samples of low [DIC] does not preclude field data interpretation. Thus, the benefits obtained from immediate sample preservation and the ability to collect more samples more frequently (due to the reduced analytical time and more portable volumes) may be preferable to having to collect larger volumes of sample to precipitate carbonate for a more precise mass spectrometric measurement of the δ^13^C_DIC_ value.

## CONCLUSIONS

Here we document a field-to-laboratory analytical procedure for the measurement of δ^13^C_DIC_ values and [DIC] for samples at the lower end of field sample DIC concentration. Our approach uses a standard matrix as similar as possible to the field samples and in doing so liquid-headspace fractionation is accommodated. Each run has its own calibration and our use of linear regression to calibrate intra-run δ^13^C_DIC_ values allows estimation of the precision (s_0_) and accuracy (95% CI) of an unknown field sample measurement. This approach is not commonplace in the community – rather repeated measurement of an internal standard, that may not even be of the same matrix, is quoted as precision, and accuracy is rarely documented.

Both mass spectrometers used here exhibit linearity effects such that for low DIC concentration samples (∼ less than 0.3 mM) our confidence in an unknown value decreases. Through recognition of analytical limits in measuring small samples of low concentrations, we have defined more rigorously the precision and accuracy with which an unknown sample composition can be measured. This precision and accuracy are calibration- and concentration-dependent, and can vary intra- and inter-run, and so should be assessed on every run – another advantage of intra-run calibration using a minimum of three standards.

The precision, s_0_, of standards and field data is comparable, such that it is difficult to assess whether the difference between standard sample preparation and field sample collection compromises the standard data and thus generates larger s_0_ values than an alternative approach. Two immediate suggestions for improvement are to adopt the automated internal standard preparation (as advocated by some authors^15^,^21^), and to temperature equilibrate the samples throughout measurement (as advocated by others^11^,^29^). Better precision on field measurements (as assessed from paired analysis) may be achieved if the exetainers are evacuated as close to use as possible, and sample volume injection can be precisely regulated (perhaps this is more difficult in a field situation). In practice our field exetainers are evacuated the day prior to field collection; thus, some air ingression may have occurred. Problems may arise where field locations are very remote, or equipment does not exist in field stations to allow the samples to be evacuated, although in these environments a hand pump may provide sufficient vacuum to check whether each exetainer has held vacuum. We suggest that triplicate field samples are collected and two of the three replicates are analysed on the same run; if the samples agree within the technique precision, analysis of the third sample is then considered unnecessary.
